# Peripheral perfusion is correlated to metabolic perfusion parameters and microvascular reactivity but not with hepatosplanchnic or microcirculatory flow parameters in hyperdynamic septic shock

**DOI:** 10.1186/cc10812

**Published:** 2012-03-20

**Authors:** G Hernandez, T Regueira, A Bruhn, P Mcnab, E Veas, C Pedreros, A Fuentealba, E Kattan, G Bugedo, M Rovegno, R Castro, C Ince

**Affiliations:** 1 Pontifi cia Universidad Catolica de Chile, Santiago, Chile; 2 University of Amsterdam, the Netherlands

## Introduction

Peripheral perfusion assessment is increasingly being recognized as a potential surrogate of global perfusion parameters during septic shock resuscitation. Nevertheless, its correlation with other perfusion parameters is not well established. This study explores the relationship between peripheral perfusion parameters and macrohemodynamic, metabolic, hepatosplanchnic, and micro-circulatory-related parameters during hyperdynamic septic shock.

## Methods

Thirty-nine sets of parallel assessments of hemodynamic or perfusion-related parameters were performed in 13 hyperdynamic (cardiac index >2.5 l/minute/m^2^) septic shock patients (age 68 ± 18 years, APACHE II score 26 ± 6, SOFA score 11 ± 4, ICU mortality 2/13) during the first 24 hours of resuscitation. Assessment included: echocardiographic and pulmonary artery catheter-derived parameters; indocyanine green plasma disappearance rate (ICG-PDR, Limon) and gastric tonometry; metabolic parameters (lactate, SvO_2 _and p(v-a) CO_2_); sublingual microcirculatory assessment (SDF); thenar StO_2 _and vascular occlusion test (VOT) derived parameters (NIRS); peripheral perfusion parameters including capillary refill time and central to toe temperature difference.

## Results

Peripheral perfusion was normal in 22 sets (56%) and abnormal in 17 (44%). A normal peripheral perfusion was associated with lower APACHE II scores (23.2 ± 3 vs. 28.4 ± 7, *P *= 0.009), better metabolic parameters (lactate: 2.3 ± 0.6 mmol/l vs. 3.5 ± 1.1 mmol/l, *P *= 0.002 and SvO_2_: 78.1 ± 6% vs. 73.9 ± 5%, *P *= 0.049), and better StO_2 _recovery slope after VOT (3.54 ± 1.4 vs. 0.94 ± 0.5%/second, *P *< 0.001) as compared with an abnormal one. No correlation could be demonstrated with macrohemodynamic parameters, hepatosplanchnic perfusion parameters (gastric tonometry and ICG-PDR), or with sublingual microcirculatory parameters.

## Conclusion

A normal peripheral perfusion is associated with normal metabolic perfusion parameters and less impaired micro-vascular reactivity. No relation between peripheral perfusion and hepatosplanchnic or sublingual microcirculatory flow could be established in this study.

## Trial Registration

ClinicalTrials.gov Identifier: NCT01271153

